# Preparation, Characterization, and Cytoprotective Effects on HUVECs of Fourteen Novel Angiotensin-I-Converting Enzyme Inhibitory Peptides From Protein Hydrolysate of Tuna Processing By-Products

**DOI:** 10.3389/fnut.2022.868681

**Published:** 2022-04-14

**Authors:** Qian-Qian Qiao, Qian-Bin Luo, Shi-Kun Suo, Yu-Qin Zhao, Chang-Feng Chi, Bin Wang

**Affiliations:** ^1^Zhejiang Provincial Engineering Technology Research Center of Marine Biomedical Products, School of Food and Pharmacy, Zhejiang Ocean University, Zhoushan, China; ^2^National and Provincial Joint Laboratory of Exploration and Utilization of Marine Aquatic Genetic Resources, National Engineering Research Center of Marine Facilities Aquaculture, School of Marine Science and Technology, Zhejiang Ocean University, Zhoushan, China

**Keywords:** skipjack tuna (*Katsuwonus pelamis*), dark muscle, angiotensin-I-converting enzyme (ACE) peptide, cytoprotective function, hypertension

## Abstract

To effectively utilize skipjack tuna (*Katsuwonus pelamis*) processing by-products to prepare peptides with high angiotensin-I-converting enzyme (ACE) inhibitory (ACEi) activity, Neutrase was selected from five kinds of protease for hydrolyzing skipjack tuna dark muscle, and its best hydrolysis conditions were optimized as enzyme dose of 1.6%, pH 6.7, and temperature of 50°C using single factor and response surface experiments. Subsequently, 14 novel ACEi peptides were prepared from the high ACEi protein hydrolysate and identified as TE, AG, MWN, MEKS, VK, MQR, MKKS, VKRT, IPK, YNY, LPRS, FEK, IRR, and WERGE. MWN, MEKS, MKKS, and LPRS displayed significantly ACEi activity with IC_50_ values of 0.328 ± 0.035, 0.527 ± 0.030, 0.269 ± 0.006, and 0.495 ± 0.024 mg/mL, respectively. Furthermore, LPRS showed the highest increasing ability on nitric oxide (NO) production among four ACEi peptides combining the direct increase and reversing the negative influence of norepinephrine (NE), and MKKS showed the highest ability on directly decreasing and reversing the side effects of NE on the secretion level of endothelin-1 (ET-1) among four ACEi peptides. These findings demonstrate that seafood by-product proteins are potential ACEi peptide sources and prepared ACEi peptides from skipjack tuna dark muscle, which are beneficial components for functional food against hypertension and cardiovascular diseases.

## Introduction

Global fish production reached around 179 million tons, in which marine capture fishery production reached about 84.4 million tons (1). However, ~50% of these catches are generated as by-products in the manufacturing process (2–4), and those by-products create burdensome disposal problems and bring serious environmental pollution problems (5–7). Therefore, researchers keep trying to establish high-value utilization methods for producing useful marketable products using functional components in fish by-products (8–12). Recently, novel bioprocessing technologies have been developed for utilizing these fish by-product proteins to prepare active peptides for commercial use (3, 13–17). Tuna and tuna-like species are commercially important marine products worldwide with 7.9 million tons of catches in 2018 (1, 18). Among them, skipjack tuna (*Katsuwonus pelamis*) is the most common and abundant species with 3.2 million tons of catches (1). During the processing of canned tuna, about 50% of skipjack tuna materials are generated as by-products (19–21). On the contrary, several functional molecules, such as collagen/gelatin, unsaturated fatty acid, protease, and polysaccharides, are generated from skipjack tuna by-products and exhibited huge application prospects and various biological activities (22, 23). Moreover, some bioactive peptides have been prepared from protein hydrolysates of tuna by-products, such as bone/frame (24), scale (25), roe (4), milt (21), and head and viscera (26, 27). In addition, Chi et al. found that protein hydrolysates of tuna dark muscle presented significant radical scavenging and lipid peroxidation inhibition ability (28). Maeda et al. revealed that the dietary tuna dark muscle could decrease hepatic steatosis and promote serum high-density lipoprotein cholesterol in obese type-2 diabetic/obese KK-A (y) mice (29).

Hypertension is taken as the most dangerous factor for cardiovascular diseases (CVD) and is closely contacted with multiple complications (30, 31). In addition, WHO reported that about 9 million people died from hypertension-related complications and accounted for about 12.8% of the world's total deaths every year, and the proportion of adults with hypertension will increase to 29.2% in 2025 (32). The angiotensin-I-converting enzyme (ACE) can convert angiotensin I (Ang I) to Ang II for inactivating the vasodilator bradykinin, and ACE inhibitory (ACEi) activity is a noticeable method for mediating systemic hypertension (33, 34). Then, chemical synthetic ACE inhibitors, such as captopril, enalapril, and lisinopril, are used as antihypertensive agents in various clinical conditions, but their untoward effects attract sustained and wild concern (31, 35). Therefore, searching for natural and safer ACEi molecules for alternative chemosynthetic drugs is vital for hypertension and CVD therapy. Currently, ACEi peptides have been prepared from a variety of marine protein resources, such as skate skin (36), mackerel skin (37), flounder fish muscle (38), smooth-hound viscera (39), Antarctic krill (34), stonefish (40), shrimp waste (41), rainbow trout viscera (42), and lizardfish (43). These ACEi peptides exhibit not only high nutritional value but also a significant biological activity for application in diet or clinical treatment on antihypertension (44–46). Therefore, the objectives of this study were to (i) optimize the preparation process of ACEi protein hydrolysate of skipjack tuna dark muscle, (ii) identify the isolated ACEi peptides, and (iii) evaluate the bioactivity of the isolated ACEi peptides.

## Materials and Methods

### Materials

Skipjack tuna (*K. pelamis*) dark muscles were provided by Ningbo Today Food Co., Ltd. (China). Sephadex G-25 resins and nitric oxide (NO) assay kits (Nitrate reductase approach, A012-1) were purchased from Nanjing Jiancheng Bioengineering Institute (China). ET-1 ELISA kit (HM10108) was purchased from Shanghai Qiaodu Biotechnology Co., Ltd. (China). Trypsin, pepsin, papain, ACE, and FAPGG were purchased from Sigma-Aldrich (Shanghai) Trading Co., Ltd. (China). Alcalase, 3-(4,5-dimethylthiazol-2yl)-2,5-diphenyltetrazolium bromide (MTT), Dulbecco's modified eagle medium (DMEM), penicillin-streptomycin liquid, dimethylsulfoxide (DMSO), fetal bovine serum (FBS), norepinephrine (NE), Neutrase and Cap were purchased from Beijing Solarbio Science & Technology Co., Ltd. (China). ACEi peptides of STAP1-STAP14 with purity higher than 95% were synthesized in Shanghai Apeptide Co., Ltd. (China).

### Preparation of Protein Hydrolysate of Skipjack Tuna Dark Muscle

#### Screening of Protease Species

The degreasing process of skipjack tuna dark muscle was performed according to the method described earlier (28). After that, defatted tuna dark muscle powders were dispersed in distilled water (1%, w/v) and hydrolyzed with an enzyme dose of 2% (w/w) using papain (55°C, pH 7.0), pepsin (37.5°C, pH 2.0), Alcalase (55°C, pH 9.5), Neutrase (55°C, pH 7.0), and trypsin (37.5°C, pH 7.8), respectively. After 3 h, the enzymolysis reaction was stopped at 95°C for 20 min and centrifuged at 8,000 × *g* for a quarter of an hour at −4°C. The resulting supernatant was lyophilized and stored at −20°C. The protein hydrolysate produced by Neutrase showed the highest ACEi activity.

#### Optimization of Hydrolysis Conditions of Neutrase

A single-factor experiment was applied to optimize the hydrolysis conditions of Neutrase. pH (8.5, 9.0, 9.5, 10, and 10.5), enzyme dose (1.0, 1.5, 2.0, 2.5, and 3.0%), and hydrolysis temperature (45, 50, 55, 60, and 65°C) were chosen for the present investigation.

According to the single-factor experiment results, response surface methodology was employed to estimate the influence of independent variables (*X*_1_, pH; *X*_2_, enzyme dose; and *X*_3_, temperature) on ACEi activity (47–49). The three levels (*X*_1_: 6.5, 7.0, and 7.5; *X*_2_: 50, 55, and 60°C; *X*_3_: 1.0, 1.5, and 2.0%) of the Box-Behnken design were designed to analyze the effects of three variables on ACEi activity. The experimental operation after hydrolysis is the same as the method described earlier. The protein hydrolysate prepared under the optimal enzymolysis conditions was referred to as TDH.

#### Separation Process of ACEi Peptides From TDH

**Figure 1:**
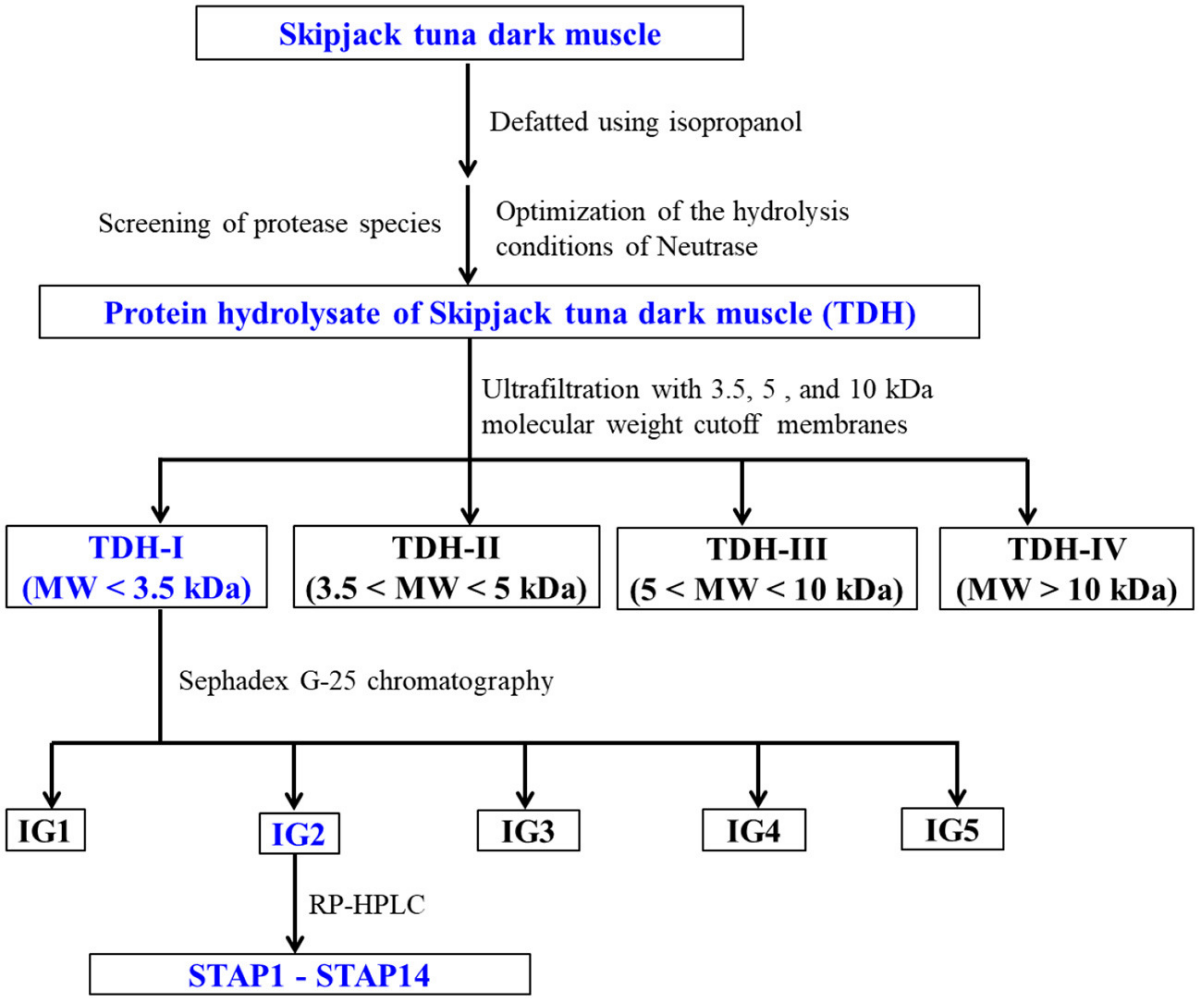
Flow diagram of purifying ACEi peptides from protein hydrolysate (TDH) of skipjack tuna (*Katsuwonus pelamis*) dark muscle prepared using Neutrase.

ACEi peptides were purified from TDH using the designed process (Figure 1).

#### Ultrafiltration

TDH (100.0 mg/ml) was fractionated with three molecular weight (MW) cutoff membranes (10, 5, and 3.5 kDa), and four peptide fractions, namely, TDH-I (<3.5 kDa), TDH-II (3.5–5 kDa), TDH-III (5–10 kDa), and TDH-IV (>10 kDa), were collected and lyophilized. TDH-I exhibited the maximum ACEi activity among the four peptide fractions.

#### Gel Permeation Chromatography

TDH-I solution (5 ml, 50.0 mg/ml) was fractionated with Sephadex G-25 column (3.6 cm × 150 cm) eluted with ultrapure water under 0.6 ml/min flow rate. Each eluate (1.8 ml) was collected by monitoring absorbance at 280 nm. Four subfractions (IG1, IG2, IG3, and IG4) were isolated from the TDH-I solution and lyophilized. The ACEi activity of IG2 was higher than those of the other three fractions.

#### Reversed-Phase High-Performance Liquid Chromatography

IG2 solution (20 μl, 100.0 μg/ml) received the final separation by RP-HPLC on a Zorbax 300SB-C18 column (9.4 mm × 250 mm, 5 μm) with a gradient of acetonitrile (1% acetonitrile in 7 min; 1–10% acetonitrile in 7 min; 10–30% acetonitrile in 7 min; 30–60% acetonitrile in 7 min; 60–100% acetonitrile in 7 min; 100 B in 5 min) inside 0.06% trifluoroacetic acid (TFA) at 2.0 ml/min flow rate. The eluate absorbance was monitored at 254 and 280 nm. A total of 14 peptides (STAP1 to STAP14) were collected according to the elution chromatogram, lyophilized, and followed by in-depth analysis for their amino acid sequences.

#### Identification of Sequence and MW of ACEi Peptide

The sequences of STAP1 to STAP14 were analyzed using an Applied Biosystems 494 protein sequencer (Perkin Elmer, USA) (9). The MWs of STAP1 to STAP14 were determined by employing a quadrupole time-of-flight (Q-TOF) mass spectrometric device (Micromass, Waters, USA) in the combination of an electrospray ionization (ESI) source (50).

#### Determination of ACEi Activity

The ACEi activity was determined by employing FAPGG as the substrate with the previous method (34). The ACEi activity was calculated directly as the decrease rate in the absorbance at 340 nm in the first 30 min ACE catalyzed hydrolysis of FAPGG. For controlled (uninhibited) reaction, the peptide sample was replaced by HEPES buffer. Half maximal inhibitory concentration (IC_50_) is the concentration of ACEi peptides required for 50% inhibition of ACE.

### Effects of STAP3, STAP4, STAP7, and STAP11 on HUVECs

#### HUVEC Culture and Cell Viability Assessment

After thawing, HUVECs were maintained in cultured flasks and cultured to confluence in DMEM containing 1% penicillin-streptomycin liquid, supplemented with 10% FBS. HUVECs received the culturing process at 37°C in a humidified 5% CO_2_ atmosphere (34).

The cytotoxicity of STAP3, STAP4, STAP7, and STAP11 on HUVECs was assessed using MTT assay on manufacturer's instructions (34). In brief, HUVECs received the incubation in 96-well plates at a density of 0.8 × 10^4^ cells per well with 180 μl completed DMEM. Following the confluence of 50–60% in the 96-well plates, cells were treated with 20 μl peptides under-designed concentrations (100, 200, and 400 μM) and further cultured for 24 h at 37°C. Subsequently, cells were added with 20 μL MTT solutions (5 mg/mL) and incubated for 4 h. Finally, DMSO was added into each well and the absorbance (A) at 490 nm was determined. The cell viability was calculated as:


$$\begin{array}{l} \text{Cell viability}\left(\%\text{ of blank control}\right)\\ =\left(A_{\text{experiment group}}/A_{\text{control group}}\right)\times 100. \end{array}$$

#### Evaluation of NO and ET-1 Production

The NO and ET-1 contents of HUVECs were determined after a 24 h treatment of ACEi peptides (STAP3, STAP4, STAP7, and STAP11, respectively) (34). HUVECs received the plating process in 6-well plates and the treating process with Cap (1 μM), NE (0.5 μM), designed concentrations of ACEi peptides (100, 200, and 400 μM) for 24 h, or treated with both NE (0.5 μM) and 200 μM ACEi peptides for 24 h. NO and ET-1 productions in treated cells were obtained by employing human NO and ET-1 assay kits according to the manufacturers' protocol.

#### Data Analysis

All data are expressed as the mean ± standard deviation (SD) with triplicate and analyzed using SPSS 20.0 (SPSS Corporation, Chicago, IL, USA). Significant differences were obtained by employing the ANOVA test with Dunnett's or Tukey's test (*P* < 0.05).

## Results and Discussion

### Preparation of Protein Hydrolysate of Skipjack Tuna Dark Muscle

#### Screening of Protease Species

Protein hydrolysates of skipjack tuna dark muscle were produced by five kinds of proteases. At 1.0 mg/ml, ACEi rate of protein hydrolysate generated by Neutrase was 66.79 ± 1.53%, which was markedly higher than the rates of protein hydrolysates produced using Alcalase (61.43 ± 2.67%), trypsin (43.19 ± 0.70%), pepsin (17.95 ± 0.44%), and papain (38.70 ± 4.65%) (*P* < 0.05). At present, several methods, including microbial fermentation, solvent extraction, enzymatic hydrolysis, and chemical degradation, have been applied to generate protein hydrolysates, and enzymatic hydrolysis is the most popular and useful process because of its easy-to-control and environmental-friendly features (3). These biological functions of protein hydrolysates have a close relationship with the composition and structure of bio-peptides, and the protease specificity is the most critical factor in the generation of bioactive protein hydrolysates (39, 44, 51). Therefore, Alcalase, Neutrase, trypsin, pepsin, papain, and their combinations are continually screened and optimized to apply in the production of protein hydrolysates from various protein resources (51–55). This study results further supported the conclusion that the specificity of proteases markedly influenced the peptide composition and biological functions of hydrolysates. Therefore, Neutrase was selected to prepare protein hydrolysate of tuna dark muscle.

#### Optimized the Hydrolysis Conditions of Neutrase Using a Single-Factor Experiment

**Figure 2:**
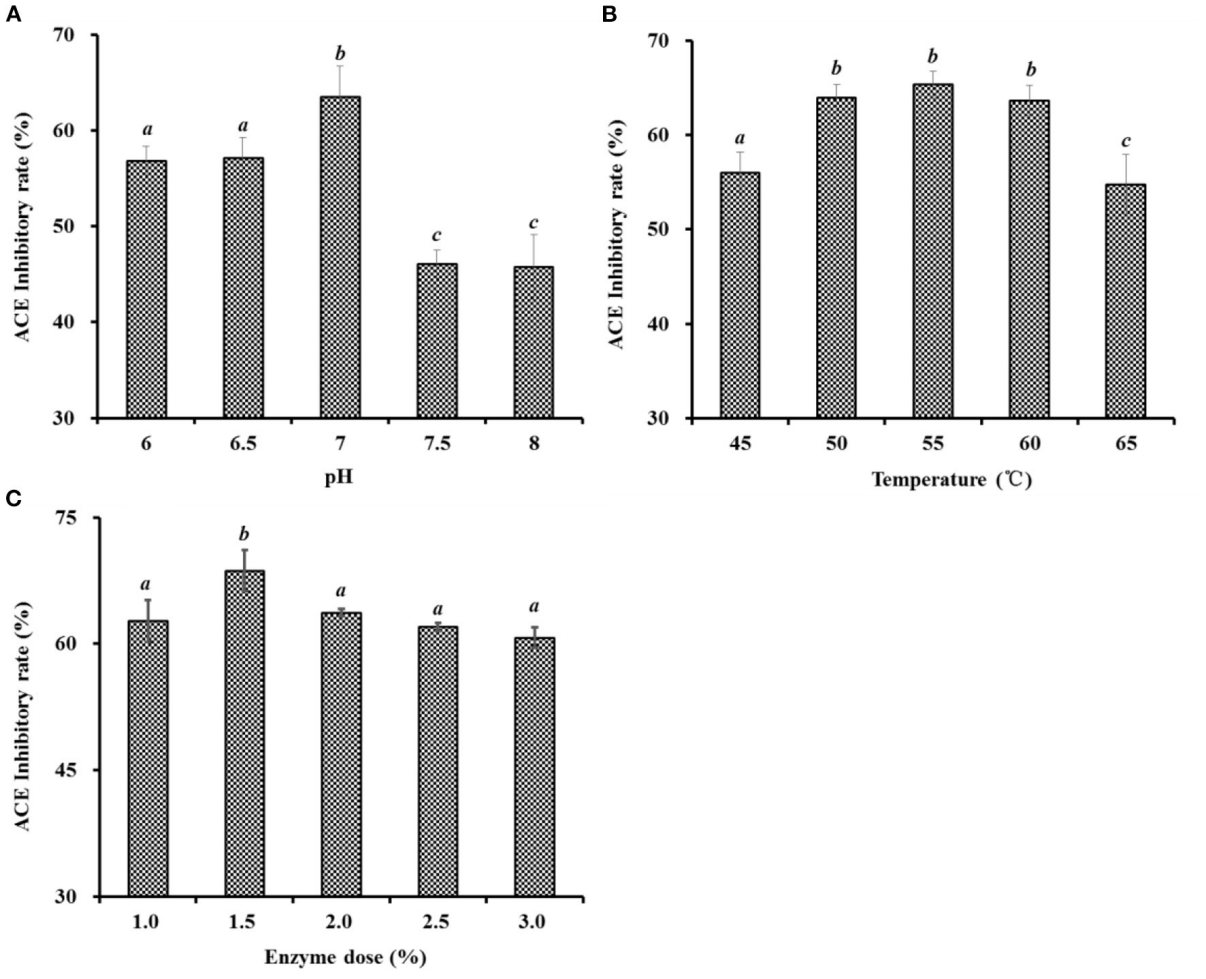
Effects of pH **(A)**, temperature (°C) **(B)**, and enzyme dose (%) **(C)** of Neutrase on ACEi activities of protein hydrolysates from skipjack tuna dark muscle at 1.0 mg/mL. ^a−*c*^Values with same letters indicate no significant difference (*P* > 0.05).

As shown in Figure 2, the effects of hydrolysis conditions of Neutrase, including pH, enzyme dose, and temperature on the ACEi activity of protein hydrolysates, were optimized by a single-factor experiment. Figure 2A indicated that pH values significantly influenced the ACEi activity of protein hydrolysates, and the ACEi activity (63.54 ± 3.19%) of protein hydrolysates prepared at pH 7.0 was prominently stronger than those of protein hydrolysate prepared at other pH values (*P* < 0.05). Figure 2B illustrated that the ACEi activity of hydrolysates increased gradually when the temperature ranged from 45 to 55°C and achieved the highest value (65.33 ± 1.42 %) at 55°C. In addition, no significant difference in ACEi activity of protein hydrolysates was observed at 50, 55, and 60°C (*P* > 0.05). Figure 2C showed that the ACEi activity of protein hydrolysates was dramatically affected by the enzyme dose, and the ACEi activity (68.64 ± 2.44%) of protein hydrolysate prepared at the dose of 1.5% was prominently stronger than those of hydrolysates prepared at other tested doses (*P* < 0.05). Additionally, the ACEi activity of protein hydrolysate was slow descent when the dose was higher than 1.5%. Therefore, the range of hydrolysis conditions for Neutrase was narrowed to 9–10, 50–60°C, and 1.0–2.0% for pH, temperature, and enzyme dose, respectively.

#### Optimized the Hydrolysis Conditions of Neutrase by Response Surface Experiment

In accordance with the results of single-factor experiment (Figure 2), the range of hydrolysis conditions for Neutrase was optimized for 6.5–7.5, 1.0–2.0%, and 50–60°C for pH (*X*_1_), enzyme dose (*X*_2_), and temperature (*X*_3_), respectively. Furthermore, the ACEi activity of protein hydrolysates under a response surface experimental design of 3-level, 3-factor factorial is shown in Table 1. After regression fitting of response values and the variables in Table 1 using Design-Expert 8.0.6, the quadratic multinomial regression equation disclosing the relationship between the ACEi rate (*Y*) and the variables (pH [*X*_1_], enzyme dose [*X*_2_], and temperature [*X*_3_]) was as given below:


$$\begin{array}{l} Y(\%)\;=\;68.55-0.86X_{1}-1.72X_{2}-1.46X_{3}-3.29X_{1}X_{2}\\ +\;1.60X_{1}X_{3}-1.51X_{2}X_{3}-3.13X_{1}^{2}-2.80X_{2}^{2}-1.08X_{3}^{2} \end{array}$$

**Table 1:** Box-Behnken design and experimental results of response surface methodology.

**Run**	**Independent variables^a^**		**Dependent variables^b^**	
	***X*** **_1_ (pH)**	***X*** **_2_(enzyme dose/%)**	***X*** **_3_(temperature/°C)**	***Y*** **(ACEi** **rate, %)**
1	6.5	1.0	55	65.65
2	6.5	1.5	60	67.43
3	7.0	2.0	60	62.80
4	7.5	2.0	55	67.17
5	7.5	1.0	55	61.99
6	6.5	2.0	55	66.19
7	7.5	1.5	60	65.40
8	7.0	1.5	55	63.78
9	7.5	1.5	50	67.09
10	7.0	1.0	50	61.55
11	7.0	1.5	55	66.97
12	6.5	1.5	50	63.70
13	7.0	1.5	55	57.11
14	7.0	2.0	50	66.77
15	7.0	1.5	55	63.91
16	7.0	1.5	55	63.52
17	7.0	1.0	60	59.55

a *Independent variables: X1, pH; X2, temperature; X3, enzyme dose*.

b *Dependent variables: Y, ACEi rate (%)*.

The results on the significance test of the coefficients of the regression model and variance analysis results of the equation are shown in Table 2, where *X*_1_, *X*_2_, *X*_3_, *X*_1_*X*_2_, *X*_1_*X*_3_, *X*_2_*X*_3_, $X_{1}^{2}$, and $X_{2}^{2}$ had significant effects on ACEi rate (*P* < 0.05). The findings made clear that the effects of variances on enzymatic hydrolysis process parameters had an interaction effect, rather than a simple linear relationship. The determination coefficient (*R*^2^) of ACEi rate was 0.9673, meaning that 96.73% of the observed results could be fitted well by this regression equation. The relationship between independent variables and the response value could be well described by the regression equation. In addition, the high degree of consistency between the predicted and observed values was proved by the adjusted determination coefficient (RAdj^2^, 0.9523), and the low variation in the mean value and high precision and good test value dependability were manifested by the low coefficient value (C.V.%, 1.49). Furthermore, the model difference was significant (*P* = 0.0002 < 0.01), and the difference of Lack of Fit (*P* = 0.6749 > 0.05) was not significant, indicating that the error of the regression equation was small and the model was well fitted. Therefore, the model could be used to analyze and predict the relationship between the ACEi rate of protein hydrolysate and various conditions of Neutrase. In addition, the analysis results in Table 2 indicate that the order of influence of each variance on ACEi rate was enzyme dose (*X*_2_) > temperature (*X*_3_) > pH (*X*_1_). Therefore, the amount of enzyme dose has a great influence on the percentage of ACE inhibition rate in the process of hydrolysis.

**Table 2:** ANOVA for response surface quadratic model: estimated regression model of the relationship between dependent variables and independent variables.

**Source**	**Sum of** **squares**	**df**	**Mean** **square**	***F*** **-Value**	***P*** **-Value**	
Model	196.17	9	21.8	23.02	0.0002	significant
*X*_1_-pH	5.92	1	5.92	6.25	0.041	
*X*_2_-E/S	23.69	1	23.69	25.02	0.0016	
*X*_3_-Temperature	17.07	1	17.07	18.03	0.0038	
*X*_1_ *X*_2_	43.39	1	43.39	45.82	0.0003	
*X*_1_ *X*_3_	10.2	1	10.2	10.78	0.0134	
*X*_2_ *X*_3_	9.16	1	9.16	9.68	0.0171	
$X_{1}^{2}$	41.13	1	41.13	43.44	0.0003	
$X_{2}^{2}$	33.12	1	33.12	34.98	0.0006	
$X_{3}^{2}$	4.88	1	4.88	5.15	0.0575	
Residual	6.63	7	0.95			
Lack of Fit	1.93	3	0.64	0.55	0.6749	Not significant
Pure Error	4.69	4	1.17			
Cor Total	202.8	16			0.0002	

**Figure 3:**
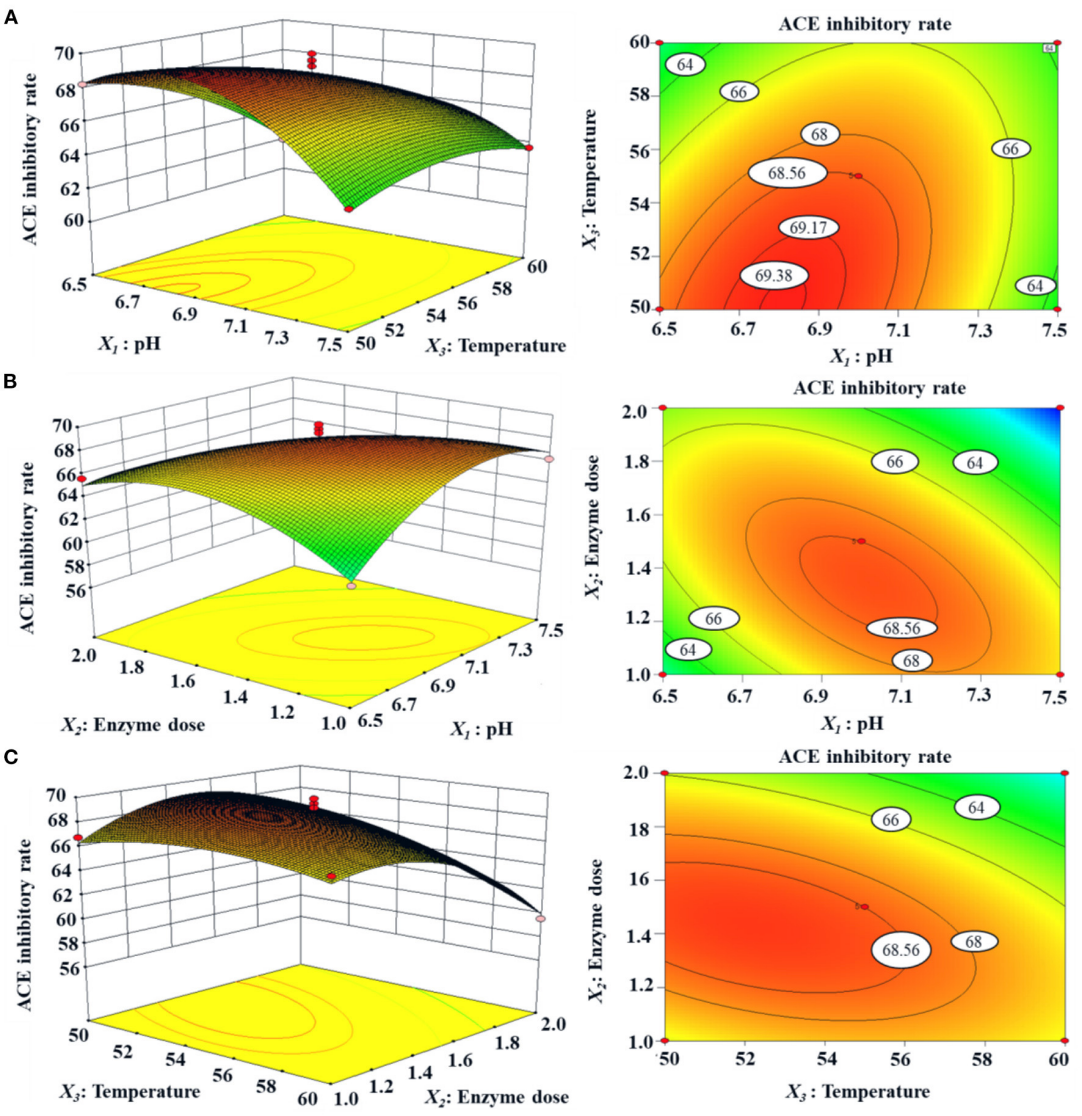
Response surface graph for ACEi rate (%) as a function of **(A)** temperature and pH, **(B)** enzyme dose and pH, **(C)** enzyme dose and temperature during the hydrolysis of skipjack tuna dark muscle with Neutrase.

The response surface diagram (Figure 3) is drawn according to the multiple nonlinear regression equation. Figure 3 is a 3D response space surface based on the interaction of response values in different experimental conditions and can predict and test the interactive influence of the variables and their mutual interaction on the ACEi rate. As shown in Figure 3A, ACEi rate increased to a certain extent when the pH value increased within a certain range, but ACEi rate decreased instead of increasing when the pH value exceeded this range. The effects of enzyme dosage and temperature showed similar trends. The shape (elliptic or round) of the contour map can reflect the strength and significance of the interaction between two independent variables. Elliptic indicates that the interaction between the two independent variables is evident; round indicates that the interaction between the two factors is not evident. The contour maps of Figures 3A–C are elliptic, indicating that the influence between the two factors (*X*_1_*X*_2_, *X*_1_*X*_3_, and *X*_2_*X*_3_) is evident. These results agreed well with the data summarized in Table 2. According to the analysis of Design-Expert 8.0.6, the optimal processing conditions of Neutrase for preparing protein hydrolysate of tuna dark muscle were as follows: pH 6.7, enzyme dosage 1.6%, and temperature 55.2°C. Using the optimum hydrolysis conditions, the ACEi rate of prepared hydrolysate (referred to as TDH) of tuna muscle was 70.96%, which was very close to the predicted 71.28% and confirmed the validity and adequacy of the predicted equation.

### Preparation of ACEi Peptides From TDH

#### Ultrafiltration

TDH underwent the fractionation into four different MW peptide fractions (TDH-I, TDH-II, TDH-III, and TDH-IV) by 3.5, 5, and 10 kDa ultrafiltration membranes. The ACEi activity of TDH-I was 51.05 ± 2.14% at 0.5 mg/ml (*P* < 0.05), which was observably higher than those of TDH (42.47 ± 1.37%), TDH-II (40.20 ± 1.52%), TDH-III (31.70 ± 1.26%), and TDH-IV (30.66 ± 1.37%). Large polypeptides are difficult to enter and bind to the ACE active site, which leads to the reduction of ACEi activity (56). Therefore, ultrafiltration technology often serves as an effective tool to collect functional peptides with low MWs from different protein hydrolysates (3, 57). The available findings were consistent with the previous reports that low MW peptide fractions of protein hydrolysates from tuna frame (35), Antarctic krill (34), rice bran (58), cooked chicken breast (56), *Cyclina sinensis* (59), and soybean (60) had the highest ACEi capabilities. Then, TDH-I with the lowest MW revealed strong ACEi capability and was selected for further separation.

#### GPC of TDH-I

**Figure 4:**
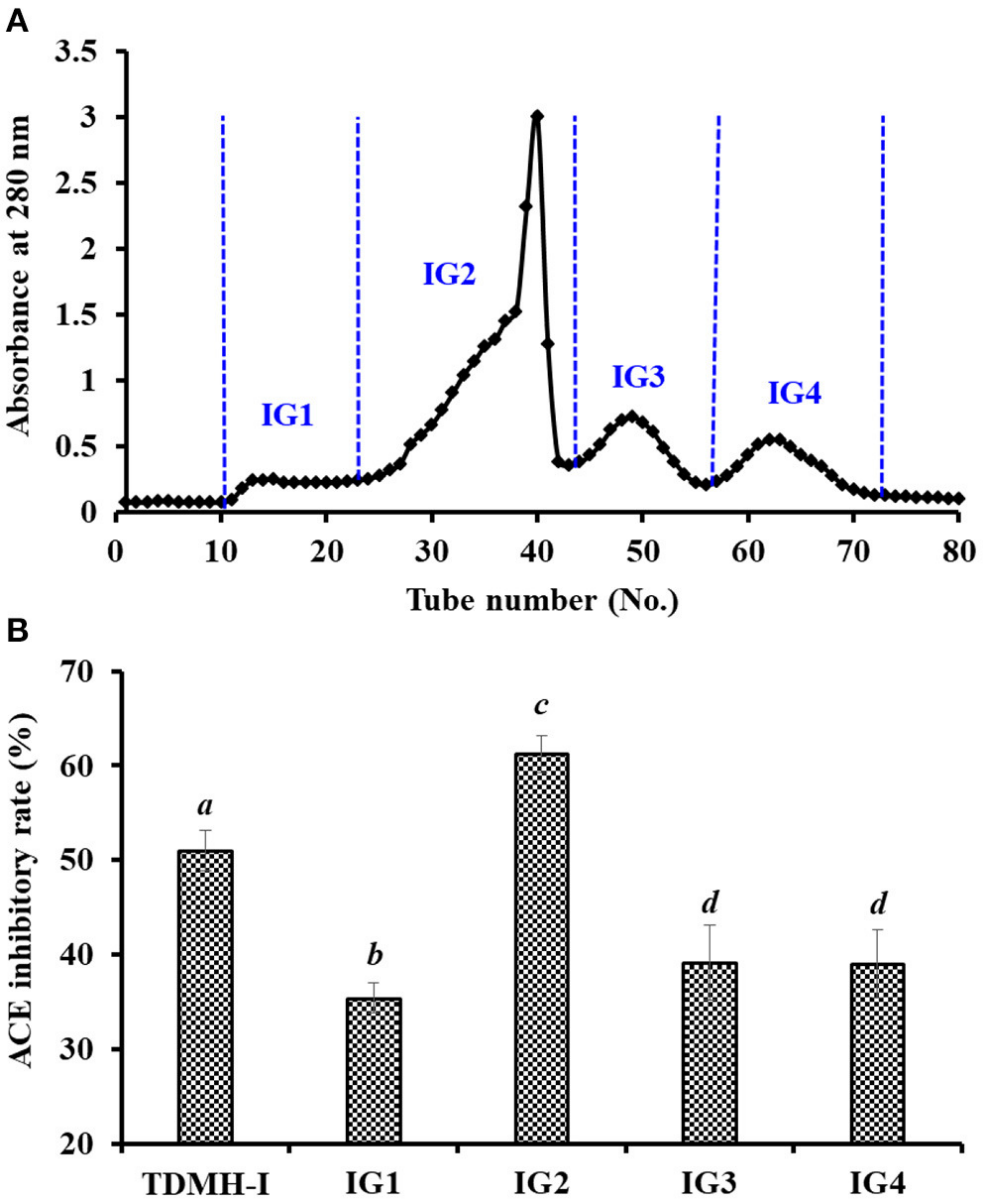
Chromatogram profiles of TDH-I isolated by Sephadex G-25 **(A)** and ACEi rates of prepared peptide fractions (IG1-IG4) from TDH-I at the concentration of 0.5 mg protein/mL **(B)**. ^a−*d*^Values with same letters indicate no significant difference (*P* > 0.05).

TDH-I was divided into four peptide fractions (IG1, IG2, IG3, and IG4) by Sephadex G-25 chromatography, and their ACEi rates are shown in Figure 4. At 0.5 mg/ml, the ACEi rate of IG2 was 61.21 ± 1.93%, which was remarkably (*P* < 0.05) higher than those of TDH-I (51.05 ± 2.14%), IG1 (35.38 ± 1.59%), IG3 (39.13 ± 3.93%), and IG4 (39.02 ± 3.61%) (Figure 4B). Gel filtration is an efficient way for purifying functional ingredients with different MW ranges and is generally applied for peptide purification from protein hydrolysates of different marine protein resources, such as skipjack tuna (27, 28), bluefin leatherjacket (52, 61), *C. sinensis* (59), miiuy croaker (9, 57), and Antarctic krill (34). Although IG2 showed the best activity, it didn't have the lowest MW among the four peptide fractions. These results suggested that other factors besides MW, such as amino acid composition and linking sequence, also greatly impact the ACEi activity of peptides (18, 46). Then, the peptide fraction of IG2 was chosen for HPLC isolation.

#### RP-HPLC Purification of IG2

**Figure 5:**
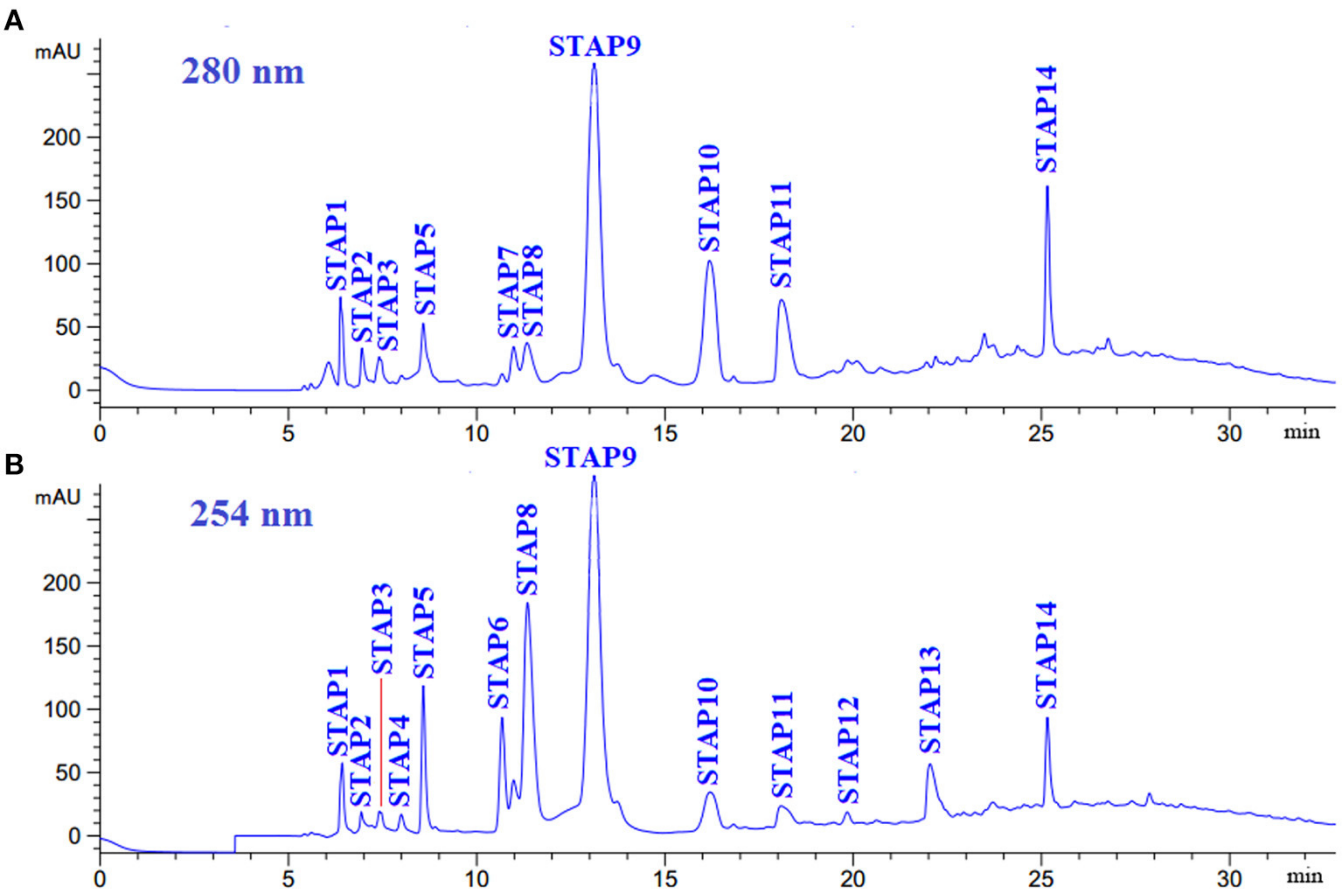
Elution profiles of subfraction IG2 by RP-HPLC using a gradient of acetonitrile containing 0.06% trifluoroacetic acid at 280 nm **(A)** and 254 nm **(B)**.

IG2 was finally purified by RP-HPLC with a Zorbax 300SB-C18 column (9.4 mm × 250 mm, 5 μm) (Figure 5). According to the elution profiles of IG2 fraction at 254 and 280 nm, 14 ACEi peptides with retention time (RT) of 6.40 min (STAP1), 6.95 min (STAP2), 7.42 min (STAP3), 8.00 min (STAP4), 8.58 min (STAP5), 10.68 min (STAP6), 10.98 min (STAP7), 11.35 min (STAP8), 13.12 min (STAP9), 16.20 min (STAP10), 18.10 min (STAP11), 19.97 min (STAP12), 22.05 min (STAP13), and 25.16 min (STAP14) were collected and lyophilized (Table 3). RP-HPLC is an extremely effective and popular technology of separation and analysis, and its RT and peak areas are devoted to qualitatively and quantitatively analyzing the isolated functional molecules (3, 62, 63). Therefore, RP-HPLC is often used for preparing ACEi peptides on their hydrophobic characters from different protein hydrolysates, such as Antarctic krill (34), rice bran (58), tuna frame (35), cooked chicken breast (56), *Ruditapes philippinarum* (64), soybean (60), stonefish (40), and smooth-hound viscera (39). Then, 14 ACEi peptides were collected and freeze-dried for further analysis.

**Table 3:** Amino acid sequences, molecular weights (MWs), and ACEi activity (IC_50_ value) of 14 isolated peptides (STAP1-STAP14) from protein hydrolysate of skipjack tuna dark muscle (TDH).

	**Retention time (min)**	**Amino acid sequence**	**Observed MW/** **Theoretical MW (Da)**	**ACEi activity (IC_**50**_, mg/mL)**
STAP1	6.40	Thr-Glu	248.24/248.23	1.885 ± 0.164^a^
STAP2	6.95	Ala-Gly	146.15/146.14	2.475 ± 0.203^b^
STAP3	7.42	Met-Trp-Asn	449.53/447.52	0.328 ± 0.035^c^
STAP4	8.00	Met-Glu-Lys-Ser	493.58/493.57	0.527 ± 0.030^c^
STAP5	8.58	Val-Lys	245.32/245.32	2.712 ± 0.492^b^
STAP6	10.68	Met-Gln-Arg	433.53/433.53	0.946 ± 0.0781^d^
STAP7	10.98	Met-Lys-Lys-Ser	492.64/492.63	0.269 ± 0.006^c^
STAP8	11.35	Val-Lys-Arg-Thr	502.60/502.61	0.868 ± 0.110^d^
STAP9	13.12	Ile-Pro-Lys	356.47/356.46	2.465 ± 0.095^b^
STAP10	16.20	Tyr-Asn-Tyr	458.47/458.46	>10^e^
STAP11	18.10	Leu-Pro-Arg-Ser	471.56/471.55	0.495 ± 0.024^c^
STAP12	19.97	Phe-Gln-Lys	421.50/421.49	1.731 ± 0.063^a^
STAP13	22.05	Ile-Arg-Arg	443.55/443.54	> 10^e^
STAP14	25.16	Trp-Glu-Arg-Gly-Glu	675.70/675.69	1.000 ± 0.123^d^

a−e *Values with same letters indicate no significant difference (P > 0.05). IC_50_ (half maximal inhibitory concentration) is the concentration of ACEi peptides required for 50% inhibition of ACE*.

#### Peptide Sequences and MWs Determination

By employing protein/peptide sequencer, sequences of 14 peptides (STAP1-STAP14) were identified as Thr-Glu (TE, STAP1), Ala-Gly (AG, STAP2), Met-Trp-Asn (MWN, STAP3), Met-Glu-Lys-Ser (MEKS, STAP4), Val-Lys (VK, STAP5), Met-Gln-Arg (MQR, STAP6), Met-Lys-Lys-Ser (MKKS, STAP7), Val-Lys-Arg-Thr (VKRT, STAP8), Ile-Pro-Lys (IPK, STAP9), Tyr-Asn-Tyr (YNY, STAP10), Leu-Pro-Arg-Ser (LPRS, STAP11), Phe-Gln-Lys (FEK, STAP12), Ile-Arg-Arg (IRR, STAP13), and Trp-Glu-Arg-Gly-Glu (WERGE, STAP14), and their MWs were determined as 248.24, 146.15, 449.53, 493.58, 245.32, 433.53, 492.64, 977.24, 356.47, 458.47, 471.56, 421.50, 443.55, and 675.70 Da, respectively, which were in good agreement with their theoretical MWs (Table 3).

### Bioactive Properties of Prepared ACEi Peptides (STAP1-STAP14)

#### ACEi Activity of Fourteen ACEi Peptides (STAP1-STAP14)

In Table 3, IC_50_ (half maximal inhibitory concentration) was used to evaluate the inhibitory efficiency of prepared peptides on ACE, and the lower of IC_50_ value indicated the more active of peptide. Therefore, STAP7 with IC_50_ value of 0.269 ± 0.006 mg/ml showed the most ACEi efficiency among 14 prepared ACEi peptides; other prepared peptides with higher ACEi activity were followed by STAP3 (0.328 ± 0.035 mg/ml), STAP11 (0.495 ± 0.024 mg/ml), and STAP4 (0.527 ± 0.030 mg/ml), respectively. In addition, the IC_50_ values of STAP3, STAP4, STAP7, and STAP11 were significantly lower than those of the other 10 ACEi peptides (>0.868 ± 0.110 mg/ml) (*P* < 0.05). However, there were no statistically significant differences among the four peptides. Additionally, the IC_50_ values of STAP3, STAP4, STAP7, and STAP11 were lower than those of ACEi peptides from protein hydrolysates of *Saurida elongate* (RYRP 3.35 mg/ml) (65), Antarctic krill (FRK: 6.97 mg/ml; YAK: 1.26 mg/ml; WRKER: 3.285 mg/ml; FQK: 1.76 mg/ml) (34), *Paralichthys olivaceus* (MEVFVP: 64.06 mg/ml; VSQLTR: 83.25 mg/ml) (38), skate (MVGSAPGVL: 3.09 mg/ml; LGPLGHQ: 4.22 mg/ml) (36), stone fish (LAPPTM: 1.31 mg/ml; EVLIQ: 1.44 mg/ml; EHPVL: 1.68 mg/ml) (40), *Okamejei kenojei* (LGPLGHQ: 3.49 mg/ml; MVGSAPGVL: 3.09 mg/ml) (66), and lizardfish (AGPPGSDGQPGA: 544.10 mg/ml) (67). The present results demonstrated that STAP3, STAP4, STAP7, and STAP11 had significantly ACEi activity and could serve as hypotensive substances applied in health products.

The molecular size determines the affinity of the peptide with ACE active sites because large peptides cannot accommodate the narrow binding channel of ACE (46). Tripeptides of VPP and IPP could easily enter into the ACE channel to bind with Zn^2+^, but larger peptides with 7–11 amino acid residues showed low-interaction scores combining with ACE active site (39). In the experiment, STAP3, STAP4, STAP7, and STAP11 belonged to tripeptide or tetrapeptide, and their small size significantly reduced the difficulty of contact with the binding channel of the ACE active site.

Peptide sequence is another key factor affecting the ACEi ability of antihypertensive peptides. Ser, Leu, and Thr at the C-terminal contribute significantly to improving the ACEi activity of KPLLCS, ELFTT, and KPLL, and this result was also supported by the data in the AHTPDB database that VFPS, MIFPGAGGPEL, PYVRYL, and IRWCT showed strong ACEi ability with IC_50_ values of 0.46, 0.03, 1.90, and 1.00 μM (56). Then, Ser residue, especially its hydroxyl group, should play a vital effect on the ACEi capability of STAP4, STAP7, and STAP11 (67). Yust et al. made clear that short peptide sequences with Met residues represent an enormous potential of ACEi peptides (68). Therefore, Met residue at the N-terminus may be one of the key factors of STAP3, STAP4, and STAP11 with high ACEi activity. Additionally, Auwal et al. illustrated that branch aliphatic or dicarboxylic amino acids (Val, Ala, Ile, and Leu) at the N-terminus could positively influence the ACEi ability of peptides (40). So, Leu residue at the N-terminus contributes to the ACEi activity of STAP11.

### Effects of STAP3, STAP4, STAP7, and STAP11 on HUVECs

#### Effects of STAP3, STAP4, STAP7, and STAP11 on Cell Viability

**Figure 6:**
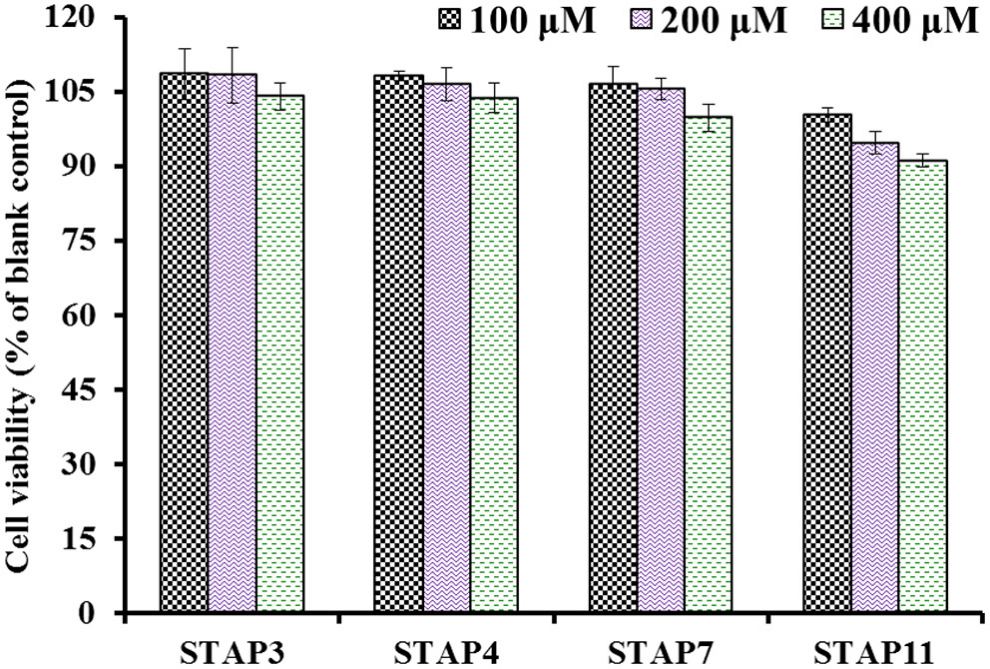
The cell viability of HUVECs treated with STAP3, STAP4, STAP7, and STAP11 for 24 h at 100, 200, and 400 μM, respectively.

The effects of STAP3, STAP4, STAP7, and STAP11 on the viability of HUVECs at the concentrations of 100–400 μM are shown in Figure 6. After incubated for 24 h at 100, 200, and 400 μM, the STAP1 group at 100 μM showed the highest cell viability (108.52 ± 5.19%), but STAP11 at 400 μM presented the lowest cell viability (91.07 ± 1.36%) among four ACEi peptides. Therefore, the cell viability of all peptide groups was higher than 90% of the blank control at the tested concentrations, which indicated that STAP3, STAP4, STAP7, and STAP11 had no significant influence on the proliferation of HUVECs (55). Vascular endothelial cells enshroud the inner surface of blood vessels and act as protective barriers within blood-vessel walls (31, 46). In addition, vascular endothelial cells serve as an active source for the metabolism of vasoactive substances and are crucial regulatory factors of vascular tone through generating vasodilatory and vasoconstrictory agents (34, 46). Then, HUVEC is an appropriate cell model for illustrating the regulating mechanism of blood pressure. In normal tissues, cells usually maintain a balance between proliferation and apoptosis, and bioactive substances with strong cell proliferation inhibition will break the balance to show their potential toxicity to tissues and are deemed unsuitable to developing non-antitumor health products (34, 69). The available results explained that STAP3, STAP4, STAP7, and STAP11 had no significant toxicity to HUVECs and should have the potential in developing antihypertensive products.

#### Effects of STAP3, STAP4, STAP7, and STAP11 on NO Production

**Figure 7:**
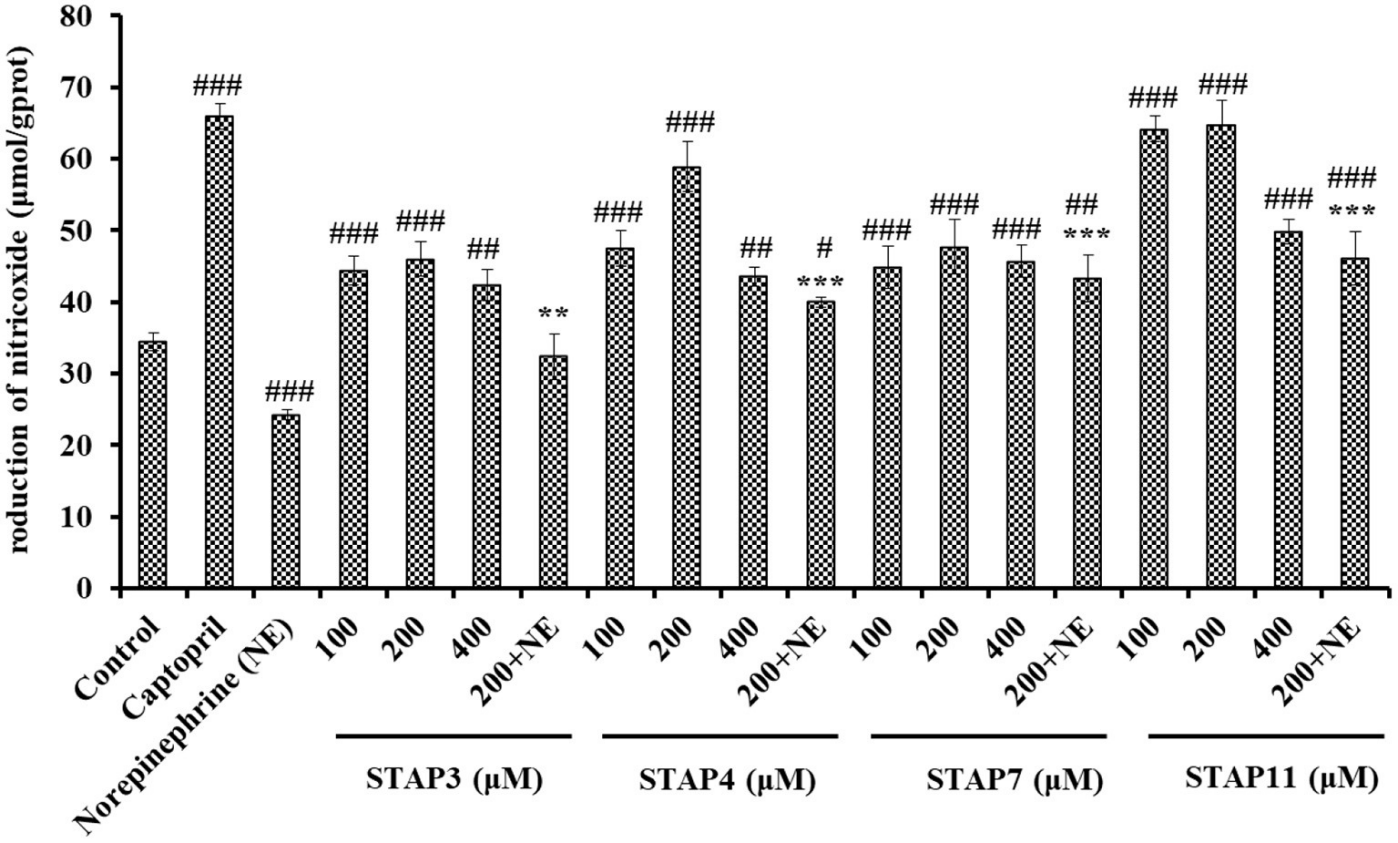
The production of nitric oxide (NO) of HUVECs treated with STAP3, STAP4, STAP7, and STAP11 for 24 h, respectively. Cell group treated with captopril (Cap) was designed as a positive control. ^###^*P* < 0.001, ^##^*P* < 0.01, and ^#^*P* < 0.05 vs. control group; ***P < 0.001 and **P < 0.01 vs. norepinephrine (NE) group.

Based on Figure 7, the NO levels in HUVECs treated with STAP3, STAP4, STAP7, and STAP11 at 100, 200, and 400 μM were significantly increased compared with the control group (34.41 ± 1.27 μmol/gprot) (*P* < 0.05), and the NO levels in HUVECs treated with STAP11 group at 100 μM reached the highest value (64.81 ± 1.79 μmol/gprot). However, the NO levels in all peptide groups were lower than that of the Cap groups (65.96 ± 1.83 μmol/gprot). Besides, NE could significantly decrease the level of NO (24.25 ± 0.63 μmol/gprot) compared with the control group (*P* < 0.001), but the negative influence of NE on reducing NO content was partially offset by STAP3, STAP4, STAP7, and STAP11 treatments at a 200-μM concentration (*P* < 0.01). Combining the direct increase and reversing the negative influence of NE, STAP11 showed the highest increasing ability on NO production among four ACEi peptides.

NO can downregulate the synthesis of Ang II type 1 receptor and ACE to antagonize the vascular tone function of angiotensin II, which further takes part in regulating the peripheral and central function of the cardiovascular system and exerts its vasoprotective effect (46). As the most potent vascular endothelium-derived vasodilator, the deficiency of NO will raise the risks of cardiovascular in pathologic situations, and the improvement of endothelial NO production represents reasonable therapeutic strategies for cardiovascular (34, 44). Previous studies proved that ACEi peptides, including TYLPVH, FQK, SP, GRVSNCAA, and VDRYF, play their antihypertensive function by increasing the NO generation of HUVECs (64, 70). Similarly, KYIPIQ can promote the generation of NO and the expression level of phosphorylated endothelial NO synthase by activating the protein kinase B (Akt) pathway in HUVECs (31). The present finding indicated that STAP3, STAP4, STAP7, and STAP11 could significantly improve the NO production in HUVECs and even reversed the NE-induced downtrend of NO production.

#### Effects of STAP3, STAP4, STAP7, and STAP11 on ET-1 Secretion

**Figure 8:**
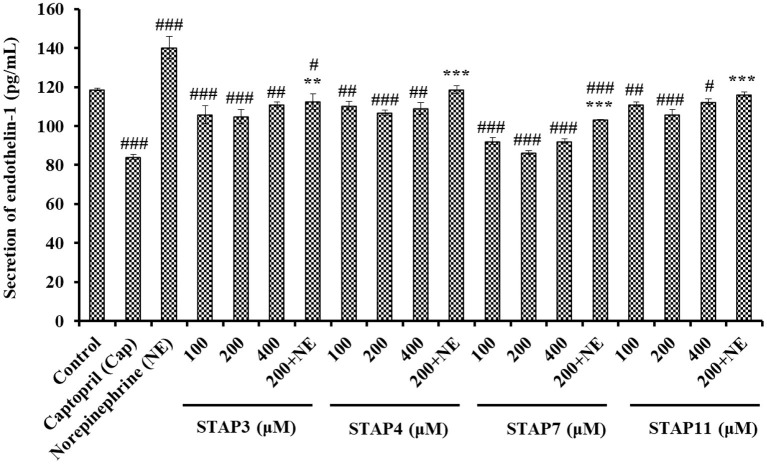
The endothelin-1 (ET-1) secretion of HUVECs treated with STAP3, STAP4, STAP7, and STAP11 for 24 h, respectively. Cell group treated with captopril (Cap) was designed as a positive control. ^###^*P* < 0.001, ^##^*P* < 0.01, and ^#^*P* < 0.05 vs. control group; ***P < 0.001 and **P < 0.01 vs. norepinephrine (NE) group.

Based on Figure 8, the ET-1 secretion of HUVECs significantly (*P* < 0.05) decreased by STAP3, STAP4, STAP7, and STAP11 under the tested concentrations, and the ET-1 level of STAP7 group reduced to 86.38 ± 0.92 pg/ml at 200 µM. Conversely, NE could significantly increase the ET-1 secretion (140.23 ± 5.81 pg/ml) compared with the control group (118.68 ± 0.53 pg/ml) (*P* < 0.01), but the ET-1 secretion affected negatively by NE was significantly (*P* < 0.01) abrogated by STAP3, STAP4, STAP7, and STAP11 treatments and decreased to 112.47 ± 3.95, 118.68 ± 1.81, 103.39 ± 0.08, and 116.15 ± 1.2 pg/ml at 200 μM (*P* < 0.01). Overall, STAP7 showed the highest ability to directly decrease and reverse the side effect of NE on the secretion level of ET-1 in HUVECs among four ACEi peptides.

As a known vasoconstriction factor, ET-1 is similar to Ang II and can lead to endothelial dysfunction related to hypertension and atherosclerosis. Previous reports confirmed that GRVSNCAA and TYLPVH from *R. philippinarum* gave play to their hypotensive activity by prominently reducing ET-1 generation (64), and the umami octapeptide of IPIPATKT could decrease the content of endothelin-1 (ET-1) in insulin-resistant-HepG2 cell and HUVECs models (71). In addition, ADVFNPR, VVLWK, LPILR, and VIEPR presented potent noncompetitive ACEi activity and significantly decreased the ET-1 levels in EA.hy926 cells (72). The available results illustrated that STAP3, STAP4, STAP7, and STAP11 displayed similar capabilities to significantly decrease the secretion of ET-1 and reversed the NE-induced uptrend of ET-1 secretion in HUVECs.

## Conclusion

The hydrolysis conditions of Neutrase for hydrolyzing the dark muscle protein of skipjack tuna were optimized, and 14 ACEi peptides were isolated from the prepared hydrolysate using Neutrase and identified as TE, AG, MWN, MEKS, VK, MQR, MKKS, VKRT, IPK, YNY, LPRS, FEK, IRR, and WERGE. MWN, MEKS, MKKS, and LPRS displayed noticeable ACEi activity. Moreover, MWN, MEKS, MKKS, and LPRS could exert their protection-related effects upon vascular endothelial functions and display an analogous antihypertensive mechanism as Cap by alleviating the negative effects of NE-constrained NO production and NE-induced ET-1 secretion. Therefore, the mentioned results provide a huge chance for skipjack tuna dark muscle as the materials to generate ACEi peptides, and the prepared ACEi peptides could serve as therapeutical ingredients to control cardiovascular disease. In addition, further study will perform the *in vivo* experiments for evaluating the therapeutic effects and illustrating the mechanisms of mentioned ACEi peptides to regulate hypertension.

## Data Availability Statement

The original contributions presented in the study are included in the article/supplementary material, further inquiries can be directed to the corresponding author/s.

## Author Contributions

Q-QQ and Q-BL contributed to data curation, methodology, and formal analysis. S-KS contributed to data curation and formal analysis. Y-QZ contributed to methodology and formal analysis. C-FC contributed to conceptualization, methodology, and supervision. BW contributed to supervision, funding acquisition, writing, reviewing, and editing. All authors contributed to the article and approved the submitted version.

## Funding

This study was funded by the National Natural Science Foundation of China (No. 82073764), Ten-thousand Talents Plan of Zhejiang Province (No. 2019R52026), and the Science and Technology Planning Project of Zhoushan of China (No. 2019C21015).

## Conflict of Interest

The authors declare that the research was conducted in the absence of any commercial or financial relationships that could be construed as a potential conflict of interest.

## Publisher's Note

All claims expressed in this article are solely those of the authors and do not necessarily represent those of their affiliated organizations, or those of the publisher, the editors and the reviewers. Any product that may be evaluated in this article, or claim that may be made by its manufacturer, is not guaranteed or endorsed by the publisher.

## Abbreviations

HUVECs: human umbilical vein endothelial cells
ACE: angiotensin-I-converting enzyme
ACEi: angiotensin-I-converting enzyme inhibitory
NO: nitric oxide
ET-1: endothelin-1
NE: norepinephrine
Cap: Captopril
Akt: protein kinase B
CVD: cardiovascular diseases
Ang: angiotensin I
MTT: 3-(4, 5-dimethylthiazol-2yl)-2, 5-diphenyltetrazolium bromide
DMEM: Dulbecco's modified eagle medium
DMSO: dimethylsulfoxide
FBSP: fetal bovine serum
TDH: protein hydrolysate of tuna dark muscle under the optimal enzymolysis conditions of Neutrase
MW: molecular weight
GPC: gel permeation chromatography
RP-HPLC: reversed-phase high-performance liquid chromatography
Q-TOF: quadrupole time-of-flight
ESI: electrospray ionization
STAP: ACEi peptide from TDH.

## References

[1] FAO. The State of World Fisheries and Aquaculture 2020. https://www.fao.org/state-of-fisheries-aquaculture/en/

[2] Li ZR Wang B Chi CF Zhang QH Gong YD Tang JJ. Isolation and characterization of acid soluble collagens and pepsin soluble collagens from the skin and bone of Spanish mackerel (*Scomberomorous niphonius*). Food Hydrocolloid. (2013) 31:103–13. 10.1016/j.foodhyd.2012.10.001

[3] Sila A Bougatef A. Antioxidant peptides from marine by-products: Isolation, identification and application in food systems. Rev J Funct Foods. (2016) 21:10–26. 10.1016/j.jff.2015.11.007

[4] Wang J Wang YM Li LY Chi CF Wang B. Twelve antioxidant peptides from protein hydrolysate of skipjack tuna (*Katsuwonus pelamis*) roe prepared by Flavourzyme: purification, sequence identification, and activity evaluation. Front Nutr. (2022) 8:813780. 10.3389/fnut.2021.813780 35127795 PMC8814634

[5] Zamora-Sillero J Gharsallaoui A Prentice C. Peptides from fish by-product protein hydrolysates and its functional properties: an overview. Mar Biotechnol. (2018) 20:118–30. 10.1007/s10126-018-9799-3 29532335

[6] Ainsa A Honrado A Marquina PL Roncalés P Beltrán JA Calanche MJB. Innovative development of pasta with the addition of fish by-products from two species. Foods. (2021) 10:1889. 10.3390/foods10081889 34441666 PMC8392829

[7] Wang WY Zhao YQ Zhao GX Chi CF Wang B. Antioxidant peptides from collagen hydrolysate of redlip croaker (*Pseudosciaena polyactis*) scales: preparation, characterization, and cytoprotective effects on H[[sb]]2[[/s]]O[[sb]]2[[/s]]-damaged HepG2 cells. Mar Drugs. (2020) 18:156. 10.3390/md18030156 32168851 PMC7142964

[8] Kim SK Mendis E. Bioactive compounds from marine processing byproducts-A review. Food Res Int. (2006) 39:383–93. 10.1016/j.foodres.2005.10.010

[9] Zhao WH Luo QB Pan X Chi CF Sun KL Wang B. Preparation, identification, and activity evaluation of ten antioxidant peptides from protein hydrolysate of swim bladders of miiuy croaker (*Miichthys miiuy*). J Funct Foods. (2018) 47:503–11. 10.1016/j.jff.2018.06.014

[10] Al Khawli F Martí-Quijal FJ Ferrer E Ruiz MJ Berrada H Gavahian M. Aquaculture and its by-products as a source of nutrients and bioactive compounds. Adv Food Nutr Res. (2020) 92:1–33. 10.1016/bs.afnr.2020.01.001 32402442

[11] Raju N Gulzar S Buamard N Ma L Ying X Zhang B. Comparative study of astaxanthin, cholesterol, fatty Acid profiles, and quality indices between shrimp oil extracted from hepatopancreas and cephalothorax. Front Nutr. (2021) 8:803664. 10.3389/fnut.2021.803664 34977134 PMC8714899

[12] Nisar U Peng D Mu Y Sun Y, A. Solution for sustainable utilization of aquaculture waste: a comprehensive review of biofloc technology and aquamimicry. Front Nutr. (2022) 8:791738. 10.3389/fnut.2021.791738 35096936 PMC8790604

[13] Wang B Li L Chi CF Ma JH Luo HY Xu YF. Purification and characterisation of a novel antioxidant peptide derived from blue mussel (*Mytilus edulis*) protein hydrolysate. Food Chem. (2013) 138:1713–9. 10.1016/j.foodchem.2012.12.002 23411302

[14] Yuan L Chu Q Wu X Yang B Zhang W Jin W. Anti-inflammatory and antioxidant activity of peptides from rthanol-soluble hydrolysates of sturgeon (*Acipenser schrenckii*) cartilage. Front Nutr. (2021) 8:689648. 10.3389/fnut.2021.689648 34179062 PMC8225940

[15] Henriques A Vázquez JA Valcarcel J Mendes R Bandarra NM Pires C. Characterization of protein hydrolysates from fish discards and by-products from the North-West Spain fishing fleet as potential sources of bioactive peptides. Mar Drugs. (2021) 19:338. 10.3390/md19060338 34199233 PMC8231949

[16] Tanzadehpanah H Asoodeh A Saberi MR Chamani J. Identification of a novel angiotensin-I converting enzyme inhibitory peptide from ostrich egg white and studying its interactions with the enzyme. Innov Food Sci Emerg. (2013) 18:212–9. 10.1016/j.ifset.2013.02.002

[17] Moosavi-Movahedi AA Hakimelahi S Chamani J Khodarahmi GA Hassanzadeh F Luo FT. Design, synthesis, and anticancer activity of phosphonic acid diphosphate derivative of adenine-containing butenolide and its water-soluble derivatives of paclitaxel with high antitumor activity. Bioorg Med Chem. (2003) 11:4303–13. 10.1016/S0968-0896(03)00524-8 13129566

[18] Yoon IS Lee G Kang SI Park SY Lee JS Kim J. Chemical composition and functional properties of roe concentrates from skipjack tuna (*Katsuwonus pelamis*) by cook-dried process. Food Sci Nutr. (2018) 6:1276–86. 10.1002/fsn3.676 30065829 PMC6060894

[19] Yu D Chi CF Wang B Ding GF Li ZR. Characterization of acid-and pepsin-soluble collagens from spines and skulls of skipjack tuna (*Katsuwonus pelamis*). Chin J Nat Med. (2014) 12:712–20. 10.1016/S1875-5364(14)60110-2 25263986

[20] Shyni K Hema GS Ninan G Mathew S Joshy CG Lakshmanan PT. Isolation and characterization of gelatin from the skins of skipjack tuna (*Katsuwonus pelamis*), dog shark (*Scoliodon sorrakowah*), and rohu (*Labeo rohita*). Food Hydrocolloid. (2014) 39:68–76. 10.1016/j.foodhyd.2013.12.008

[21] Wang YM Li XY Wang J He Y Chi CF Wang B. Antioxidant peptides from protein hydrolysate of skipjack tuna milt: Purification, identification, and cytoprotection on H[[sb]]2[[/s]]O[[sb]]2[[/s]] damaged human umbilical vein endothelial cells. Process Biochem. (2022) 113:258–69. 10.1016/j.procbio.2022.01.008

[22] Klomklao S Kishimura H Nonami Y Benjakul S. Biochemical properties of two isoforms of trypsin purified from the Intestine of skipjack tuna (*Katsuwonus pelamis*). Food Chem. (2009) 115:155–62. 10.1016/j.foodchem.2008.11.087

[23] Kim YM. Kim EY Kim IH Nam TJ. Peptide derived from desalinated boiled tuna extract inhibits adipogenesis through the downregulation of C/EBP-α and PPAR-γ in 3T3-L1 adipocytes. Int J Mol Med. (2015) 35:1362–8. 10.3892/ijmm.2015.2127 25761066

[24] Yang XR Zhao YQ Qiu YT Chi CF Wang B. Preparation and characterization of gelatin and antioxidant peptides from gelatin hydrolysate of skipjack tuna (*Katsuwonus pelamis*) bone stimulated by *in vitro* gastrointestinal digestion. Mar Drugs. (2019) 17:78. 10.3390/md17020078 30678362 PMC6410064

[25] Qiu YT Wang YM Yang XR Zhao YQ Chi CF Wang B. Gelatin and antioxidant peptides from gelatin hydrolysate of skipjack tuna (*Katsuwonus pelamis*) scales: preparation, identification and activity evaluation. Mar Drugs. (2019) 17:565. 10.3390/md17100565 31623339 PMC6836156

[26] Martínez-Alvarez O Batista I Ramos C Montero P. Enhancement of ACE and prolyl oligopeptidase inhibitory potency of protein hydrolysates from sardine and tuna by-products by simulated gastrointestinal digestion. Food Funct. (2016) 7:2066–73. 10.1039/C5FO01603G 27045751

[27] Zhang L Zhao GX Zhao YQ Qiu YT Chi CF Wang B. Identification and active evaluation of antioxidant peptides from protein hydrolysates of skipjack tuna (*Katsuwonus pelamis*) head. Antioxidants. (2019) 8:318. 10.3390/antiox8080318 31430875 PMC6721175

[28] Chi CF Hu FY Wang B Li ZR Luo HY. Influence of amino acid compositions and peptide profiles on antioxidant capacities of two protein hydrolysates from skipjack tuna (*Katsuwonus pelamis*) dark muscle. Mar Drugs. (2015) 13:2580–601. 10.3390/md13052580 25923316 PMC4446595

[29] Maeda H Hosomi R Fukuda M Ikeda Y Yoshida M Fukunaga K. Dietary tuna dark muscle protein attenuates hepatic steatosis and increases serum high-density lipoprotein cholesterol in obese type-2 diabetic/obese KK-A(y) mice. J Food Sci. (2017) 82:1231–8. 10.1111/1750-3841.13711 28422289

[30] Lammi C Aiello G Boschin G Arnoldi A. Multifunctional peptides for the prevention of cardiovascular disease: A new concept in the area of bioactive food-derived peptides. J Funct Foods. (2019) 55:135–45. 10.1016/j.jff.2019.02.016

[31] Lin K Ma Z Ramachandran M De Souza C Han X Zhang L. inhibitory peptide KYIPIQ derived from yak milk casein induces nitric oxide production in HUVECs and diffuses *via* a transcellular mechanism in Caco-2 monolayers. Process Biochem. (2020) 99:103–11. 10.1016/j.procbio.2020.08.031

[32] Liu X Li F Zheng Z Li G Zhou H Zhang T. Association of morning hypertension with chronic kidney disease progression and cardiovascular events in patients with chronic kidney disease and hypertension. Nutr Metab Cardiovas. In press. (2022). 10.1016/j.numecd.2021.12.021 35172934

[33] Ju DT. K AK Kuo WW Ho TJ Chang RL Lin WT. Bioactive peptide VHVV upregulates the long-term memory-related biomarkers in adult spontaneously hypertensive rats. Int J Mol Sci. (2019) 20:3069. 10.3390/ijms20123069 31234585 PMC6627188

[34] Zhao YQ Zhang L Tao J Chi CF Wang B. Eight antihypertensive peptides from the protein hydrolysate of Antarctic krill (*Euphausia superba*): Isolation, identification, and activity evaluation on human umbilical vein endothelial cells (HUVECs). Food Res Int. (2019) 121:197–204. 10.1016/j.foodres.2019.03.035 31108740

[35] Lee SH Qian ZJ Kim SK, A. novel angiotensin I converting enzyme inhibitory peptide from tuna frame protein hydrolysate and its antihypertensive effect in spontaneously hypertensive rats. Food Chem. (2010) 118:96–102. 10.1016/j.foodchem.2009.04.086

[36] Ngo DH Ryu B Kim SK. Active peptides from skate (*Okamejei kenojei*) skin gelatin diminish angiotensin-I converting enzyme activity and intracellular free radical-mediated oxidation. Food Chem. (2014) 143:246–55. 10.1016/j.foodchem.2013.07.067 24054237

[37] Khiari Z Rico D Martin-Diana AB Barry-Ryan C. Structure elucidation of ACE-inhibitory and antithrombotic peptides isolated from mackerel skin gelatine hydrolysates. J Sci Food Agric. (2014) 94:1663–71. 10.1002/jsfa.6476 24214841

[38] Ko JY Kang N Lee JH Kim JS Kim WS Park SJ. Angiotensin I-converting enzyme inhibitory peptides from an enzymatic hydrolysate of flounder fish (*Paralichthys olivaceus*) muscle as a potent anti-hypertensive agent. Process Biochem. (2016) 51:535–41. 10.1016/j.procbio.2016.01.009

[39] Abdelhedi O Nasri R Mora L Jridi M Toldra F Nasri M. In silico analysis and molecular docking study of angiotensin I-converting enzyme inhibitory peptides from smooth-hound viscera protein hydrolysates fractionated by ultrafiltration. Food Chem. (2018) 239:453–63. 10.1016/j.foodchem.2017.06.112 28873590

[40] Auwal SM Abidin NZ Zarei M Tan CP Saari N. Identification, structure-activity relationship and in silico molecular docking analyses of five novel angiotensin I-converting enzyme (ACE)-inhibitory peptides from stone fish (*Actinopyga lecanora*) hydrolysates. PLoS ONE. (2019) 14:e0197644. 10.1371/journal.pone.0197644 31145747 PMC6542528

[41] Joshi I Janagaraj K Noorani KPM Nazeer RA. Isolation and characterization of angiotensin I-converting enzyme (ACE-I) inhibition and antioxidant peptide from by-catch shrimp (*Oratosquilla woodmasoni*) waste. Biocatal Agric Biotechnol. (2020) 29:101770. 10.1016/j.bcab.2020.101770

[42] Vásquez P. Zapata JE Chamorro VC Fillería SFG Tironi VA. Antioxidant and angiotensin I-converting enzyme (ACE) inhibitory peptides of rainbow trout (*Oncorhynchus mykiss*) viscera hydrolysates subjected to simulated gastrointestinal digestion and intestinal absorption. LWT. (2022) 154:112834. 10.1016/j.lwt.2021.112834

[43] Lu Y Wu Y Hou X Lu Y Meng H Pei S. Separation and identification of ACE inhibitory peptides from lizard fish proteins hydrolysates by metal affinity-immobilized magnetic liposome. Protein Expres Purif. (2022) 191:106027. 10.1016/j.pep.2021.106027 34838725

[44] Abdelhedi O Nasri M. Basic and recent advances in marine antihypertensive peptides: Production, structure-activity relationship and bioavailability. Trends Food Sci Technol. (2019) 88:43–557. 10.1016/j.tifs.2019.04.002

[45] Abachi S Bazinet L Beaulieu L. Antihypertensive and angiotensin-I-converting enzyme (ACE)-inhibitory peptides from fish as potential cardioprotective compounds. Mar Drugs. (2019) 17:613. 10.3390/md17110613 31671730 PMC6891548

[46] Fan H Liao W Wu J. Molecular interactions, bioavailability, and cellular mechanisms of angiotensin-converting enzyme inhibitory peptides. J Food Biochem. (2019) 43:e12572. 10.1111/jfbc.12572 31353484

[47] Fang X Xie N Chen X Yu H Chen J. Optimization of antioxidant hydrolysate production from flying squid muscle protein using response surface methodology. Food Bioprod Process. (2012) 90:676–82. 10.1016/j.fbp.2012.04.001

[48] Wang D Shahidi F. Protein hydrolysate from turkey meat and optimization of its antioxidant potential by response surface methodology. Poultry Sci. (2018) 97:1824–31. 10.3382/ps/pex457 29471508

[49] Korkmaz K Tokur B. Optimization of hydrolysis conditions for the production of protein hydrolysates from fish wastes using response surface methodology. Food Biosci. (2022) 45:101312 10.1016/j.fbio.2021.101312

[50] Chi CF Wang B Wang YM Deng SG Ma JY. Isolation and characterization of three antioxidant pentapeptides from protein hydrolysate of monkfish (*Lophius litulon*) muscle. Food Res Int. (2014) 55:222–8. 10.1016/j.foodres.2013.11.018

[51] Wang B Wang YM Chi CF Luo HY Deng SG Ma JY. Isolation and characterization of collagen and antioxidant collagen peptides from scales of croceine croaker (*Pseudosciaena crocea*). Mar Drugs. (2013) 11:4641–61. 10.3390/md11114641 24284428 PMC3853751

[52] Chi CF Wang B Hu FY Wang YM Zhang B Deng SG. Purification and identification of three novel antioxidant peptides from protein hydrolysate of bluefin leatherjacket (*Navodon septentrionalis*) skin. Food Res Int. (2015) 73:124–9. 10.1016/j.foodres.2014.08.038

[53] Zhao GX Yang XR Wang YM Zhao YQ Chi CF Wang B. Antioxidant peptides from the protein hydrolysate of Spanish mackerel (*Scomberomorous niphonius*) muscle by *in vitro* gastrointestinal digestion and their in vitro activities. Mar Drugs. (2019) 17:531. 10.3390/md17090531 31547415 PMC6780850

[54] Yang XR Qiu YT Zhao YQ Chi CF Wang B. Purification and characterization of antioxidant peptides derived from protein hydrolysate of the marine bivalve mollusk *Tergillarca granosa*. Mar Drugs. (2019) 17:251. 10.3390/md17050251 31035632 PMC6563033

[55] Wang YZ Zhao YQ Wang YM Zhao WH Wang P Chi CF. Antioxidant peptides from Antarctic Krill (*Euphausia superba*) hydrolysate: Preparation, identification and cytoprotection on H[[sb]]2[[/s]]O[[sb]]2[[/s]]-induced oxidative stress. J Funct Foods. (2021) 86:104701. 10.1016/j.jff.2021.104701

[56] Sangsawad P Roytrakul S Yongsawatdigul J. Angiotensin converting enzyme (ACE) inhibitory peptides derived from the simulated *in vitro* gastrointestinal digestion of cooked chicken breast. J Funct Foods. (2017) 29:77–83. 10.1016/j.jff.2016.12.005 29426427

[57] He Y Pan X Chi CF Sun KL Wang B. Ten new pentapeptides from protein hydrolysate of miiuy croaker (*Miichthys miiuy*) muscle: Preparation, identification, and antioxidant activity evaluation. LWT. (2019) 105:1–8. 10.1016/j.lwt.2019.01.054

[58] Wang X Chen H Fu X Li S Wei J, A. novel antioxidant and ACE inhibitory peptide from rice bran protein: Biochemical characterization and molecular docking study. LWT. (2017) 75:93–9. 10.1016/j.lwt.2016.08.047

[59] Yu F Zhang Z Luo L Zhu J Huang F Yang Z. Identification and molecular docking study of a novel angiotensin-I converting enzyme inhibitory peptide derived from enzymatic hydrolysates of *Cyclina sinensis*. Mar Drugs. (2018) 16:411. 10.3390/md16110411 30373231 PMC6265983

[60] Xu Z Wu C Sun-Waterhouse D Zhao T Waterhouse GIN Zhao M. Identification of post-digestion angiotensin-I converting enzyme (ACE) inhibitory peptides from soybean protein Isolate: Their production conditions and *in silico* molecular docking with ACE. Food Chem. (2021) 345:128855. 10.1016/j.foodchem.2020.128855 33340899

[61] Chi CF Wang B Wang YM Zhang B Deng SG. Isolation and characterization of three antioxidant peptides from protein hydrolysate of bluefin leatherjacket (*Navodon septentrionalis*) heads. J Funct Foods. (2015) 12:1–10. 10.1016/j.jff.2014.10.027

[62] Hu XM Wang YM Zhao YQ Chi CF Wang B. Antioxidant Peptides from the Protein Hydrolysate of Monkfish (*Lophius litulon*) Muscle: Purification, Identification, and Cytoprotective Function on HepG2 Cells Damage by H[[sb]]2[[/s]]O[[sb]]2[[/s]]. Mar Drugs. (2020) 18:153. 10.3390/md18030153 32164197 PMC7142609

[63] Chi CF Hu FY Wang B Li T Ding GF. Antioxidant and anticancer peptides from the protein hydrolysate of blood clam (*Tegillarca granosa*) muscle. J Funct Foods. (2015) 15:301–13. 10.1016/j.jff.2015.03.045

[64] Zhang Y Pan Da Yang Z Gao X Dang Y. Angiotensin I-Converting enzyme (ACE) inhibitory and dipeptidyl Peptidase-4 (DPP-IV) inhibitory activity of umami peptides from *Ruditapes philippinarum*. LWT. (2021) 144:111265. 10.1016/j.lwt.2021.111265

[65] Sun L Wu S Zhou L Wang F Lan X Sun J. Separation and characterization of Angiotensin I Converting Enzyme (ACE) inhibitory peptides from *Saurida elongata* proteins hydrolysate by IMAC-Ni^2+^. Mar Drugs. (2017) 15:29–39. 10.3390/md15020029 28212269 PMC5334609

[66] Ngo DH Kang KH Ryu B Vo TS Jung WK Byun HG. Angiotensin-I converting enzyme inhibitory peptides from antihypertensive skate (*Okamejei kenojei*) skin gelatin hydrolysate in spontaneously hypertensive rats. Food Chem. (2015) 174:37–43. 10.1016/j.foodchem.2014.11.013 25529649

[67] Chen J Liu Y Wang G Sun S Liu R Hong B. Processing optimization and characterization of angiotensin-?-converting enzyme inhibitory peptides from lizardfish (*Synodus macrops*) scale gelatin. Mar Drugs. (2018) 16:228. 10.3390/md16070228 29973522 PMC6071053

[68] Yust MM Pedroche J Calle JG Alaiz M Millán F Vioque J. Production of ace inhibitory peptides by digestion of chickpea legumin with alcalase. Food Chem. (2003) 81:363–9. 10.1016/S0308-8146(02)00431-4

[69] Cai SY Wang YM Zhao YQ Chi CF Wang B. Cytoprotective effect of antioxidant pentapeptides from the protein hydrolysate of swim bladders of miiuy croaker (Miichthys miiuy) against H[[sb]]2[[/s]]O[[sb]]2[[/s]]-mediated human umbilical vein endothelial cell (HUVEC) injury. Int J Mol Sci. (2019) 20:5425. 10.3390/ijms20215425 31683554 PMC6862189

[70] Zheng SL Luo QB Suo SK Zhao YQ Chi CF Wang B. Preparation, identification, molecular docking study and protective function on HUVECs of novel ACE inhibitory peptides from protein hydrolysate of skipjack tuna muscle. Mar Drugs. (2022) 20:176. 10.3390/md20030176 35323475 PMC8954214

[71] Chen M Pan D Zhou T Gao X Dang Y. Novel umami peptide IPIPATKT with dual dipeptidyl peptidase-IV and angiotensin I-converting enzyme inhibitory activities. J Agric Food Chem. (2021) 69:5463–70. 10.1021/acs.jafc.0c07138 33949854

[72] Zheng Y Li Y Zhang Y Ruan X Zhang R. Purification, characterization, synthesis, *in vitro* ACE inhibition and in vivo antihypertensive activity of bioactive peptides derived from oil palm kernel glutelin-2 hydrolysates. J Funct Foods. (2017) 28:48–58. 10.1016/j.jff.2016.11.021

